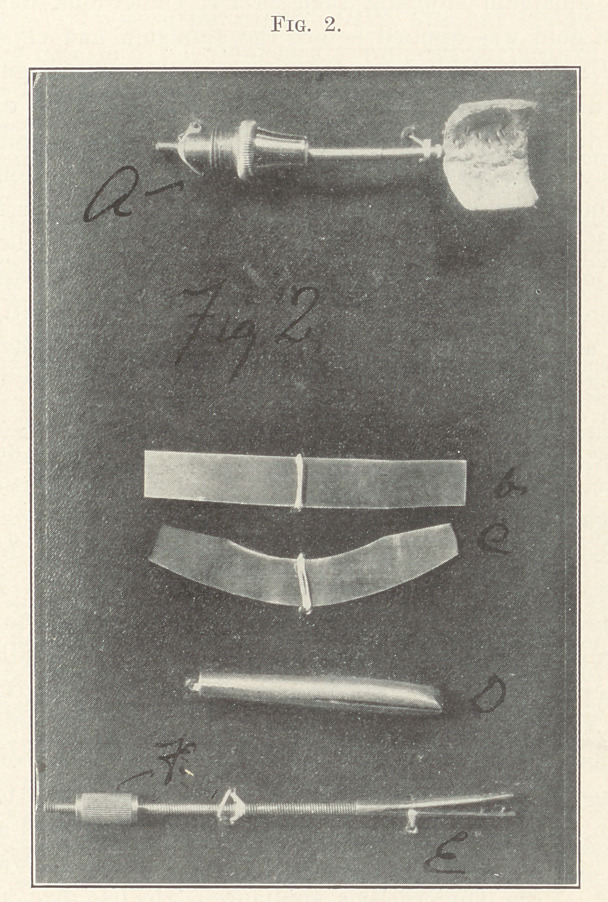# The New York Institute of Stomatology

**Published:** 1904-05

**Authors:** 

**Affiliations:** The New York Institute of Stomatology


### Reports of Society Meetings.

### THE NEW YORK INSTITUTE OF STOMATOLOGY.

A MEETING of the Institute was held at the "Chelsea," No. 222 West Twenty-third Street, New York, on Tuesday, January 6, 1903, the President, Dr. J. Morgan Howe, in the chair.

The minutes of the previous meeting were read and approved.

On account of the lateness of the hour, the communications on theory and practice were omitted. Dr. H. A. Baker, of Boston, Mass., read a paper entitled "Treatment of Protruding and Receding Jaws by the Use of the Intermaxillary Elastics."

(For Dr. Baker's paper, see page 344.)

### DISCUSSION.

Dr. C. F. Allen said that, in considering the beautiful work we have seen to-day, as the result of Dr. Baker's methods, it is very hard to differentiate and say how much credit belongs to Dr. Baker, and how much to Dr. Angle. Both of the gentlemen have been working along similar lines; that is, Dr. Baker uses the expansion arch connected with bands fastened to the back teeth as advocated and practised by Dr. Angle, and he in no way antagonizes Dr. Angle's methods or apparatus; *per contra,* Dr. Baker has given us his system of intermaxillary elastics, and these are used in connection with the expansion arch as well by Dr. Angle as by Dr. Baker.

The reciprocal action of these intermaxillary elastics designed by Dr. Baker is perfect; there is absolutely no waste force, and those difficult cases of protruding upper jaws which have been to us all such a serious problem are now made comparatively easy, and the time necessary to bring about the reduction of this malocclusion is easily lessened from fifty to seventy-five per cent. All praise to Dr. Baker! It almost starts a new epoch in orthodontia.

There was present this afternoon at the informal exhibition of patients at Dr. Kimball's office a distinguished member of our profession who has probably done more of this kind of work than any of us. I refer to Dr. Kingsley, and he, looking at the patient

I had in hand, and praising greatly the quick and beautiful results attained by Dr. Baker's methods, said to me, "*Now keep them there.*" He knew what he was saying, and his adjuration to me was born of large experience; and here again comes in the beauty of this intermaxillary combination,—it makes in many ways an ideal retaining device, and it is with its aid that I hope to keep these teeth and these jaws in their proper position.

Dr. E. A. Bogue said that between Dr. Angle and Dr. Baker we were learning a great deal about regulating. Dr. Allan spoke of the "angle appliance." Dr. Bogue's impression was that this went back as far as the "Kingsley appliance," and maybe still farther back. Dr. Allan also mentioned the serious problem of retaining teeth. Dr. Bogue was pleased to hear again from Dr. Baker, regarding his son's case, that owing to lack of time he had been unable to make a retaining appliance, and so left the regulating fixture in place, which made the best possible retaining appliance.

Dr. Bogue said that Dr. Baker did not refer to malocclusion with anything like the emphasis that he might have done. Because upon that point retention depended very largely. The teeth get out of position for various reasons, but when once replaced, the cusps of the occluding teeth would hold them in place. When both arches have been regulated, if the lower teeth are properly retained, we need not bother about the upper ones, if the occlusion is right.

Dr. Bogue believed that regulating should be commenced as soon as the permanent first molars were sufficiently developed to hold an appliance.

Dr. Baker had shown us a case where he had stated that the chin, by actual measurement, was a quarter of an inch farther back after he had finished with it. Dr. Bogue thought if he would consider the relation of the molars and bicuspids, upper and lower, before and after the regulating, and then take into consideration the nature of the hinge of the inferior maxilla, this apparent retraction of the chin would be accounted for. The lower jaw had really been dropped and the chin was consequently retracted. The work Dr. Baker was engaged in was intensely interesting, and it was work that claimed all that was best in a man.

Dr. N. W. Kingsley spoke of seeing at Dr. Kimball's office a case treated by Dr. Allan, according to Dr. Baker's method, and said that he had never seen so ingenious an appliance for this purpose. Dr. Kingsley had regulated many similar cases in past years, but by entirely different methods; this plan of Dr. Baker's was so much more simple and quite as effective, that Dr. Kingsley said that he envied Dr. Baker the credit for having invented it.

Dr. Kingsley added that his greatest satisfaction at this time was, that the question of jumping the bite seemed to have been universally accepted and settled. He believed that he was the first person who ever adopted and carried out that principle of correcting malocclusion of the jaws, and that the term originated with him. That twenty-five years or more ago he had corrected a case in a very brief space of time, and was exhibiting models of the result at a meeting of the Odontological Society. Some one asked how it was done, and Dr. Kingsley answered, on the spur of the moment, that he "jumped the bite." The phrase was taken up, the method discussed, and the facts disputed for many years, notably by Dr. Talbot, of Chicago. Therefore all the gentlemen could appreciate the pleasure it gave him to see that the principles had not only been accepted, but the method of accomplishing it improved upon by Dr. Baker.

Dr. Ashley Faught would like to ask Dr. Baker a little more explicitly regarding the bands he used, and also as to the guide of tension and pressure. Dr. Faught used what are known as election bands, although he had some a little larger in circumference and a little thinner made for him by the Goodyear Rubber Co. His guide of tension was what pressure could be comfortably stood by the patient.

Dr. Geo. S. Allan would like to ask Dr. Baker or Dr. Kingsley to more clearly define just what changes take place in this so-called "jumping the bite."

Dr. Kingsley did not believe any one could tell.

Dr. C. F. Allen said, in relation to this question, that Dr. McBride delivered a paper before the Association of American Dentists, in Europe, upon this subject. He thinks there is a change in the articulation. In a letter that Dr. Allen had recently received from Dr. McBride, he stated that he was going to make some experiments upon monkeys and then kill the monkeys, with a view to obtaining information upon this subject.

Dr. Baker, in closing, said that he had been criticised many times for regulating teeth too fast, but he thought that when we know just where we are going to move them, the teeth could not be moved too rapidly. The teeth always gave a timely warning if it were necessary to relieve the pressure. The fact that he made no false steps would account for some of the rapidity with which he completed the work. Two months was the rule in cases of anterior protrusion.

Dr. Bogue seemed to think he did not put stress enough upon the bad effects of extraction. Dr. Baker thought the cases calling for extraction were very rare, indeed, if they ever occurred. He believed that in fifteen years he had not extracted a tooth for regulating purposes.

It was a point of great satisfaction to him that Dr. Kingsley could be present, and he was much gratified at his favorable criticisms. The profession were greatly indebted to Dr. Kingsley.

It was moved and seconded that a vote of thanks be extended to Dr. Baker for his excellent paper and his kindness in demonstrating this subject so carefully. The thanks of the Institute were also extended to Dr. Baker's patient.

At the afternoon clinic the following case was presented by Dr. C. F. Allen:

Master W. H., aged eleven years, a case of protruding upper incisors with receding lower jaw; lower front teeth elongated and impinging on palatal surface of upper jaw.

Treated solely from within the mouth with intermaxillary elastics, as suggested by Dr. Baker, of Boston.

First elastics put on November 14, and case practically finished December 20, the protruding upper teeth in proper position, the anterior lower teeth shortened in their sockets and in proper relation with the upper teeth; both jaws in proper occlusion.

Present fixture used for the correction of the malocclusion will probably be the only retaining device used.

Adjourned.

---

The annual meeting of The New York Institute of Stomatology was held on Tuesday evening, December 1, 1903, and the following officers were elected for 1904:

President, A. H. Brockway; Vice-President, C. O. Kimball; Recording Secretary, H. L. Wheeler; Corresponding Secretary, J. B. Locherty; Treasurer, J. A. Bishop; Editor, F. L. Bogue; Curator, S. H. McNaughton.

*Executive Committee.*—S. E. Davenport, Chairman, C. F. Allen, F. Milton Smith.

---

A regular meeting of The New York Institute of Stomatology was held at the "Chelsea," No. 222 West Twenty-third Street, New York, on Tuesday evening, January 5, 1904, the President, Dr. Brockway, in the chair.

The minutes of the last regular meeting and of the annual meeting were read and approved.

Under the head of "Practical Talks on Interesting Subjects," Dr. S. F. Howland discussed the subject of sensitive necks of teeth.

Dr. Howland stated that we frequently found at the neck of a tooth extreme sensitiveness, which condition was very annoying to both patient and operator. Not very much had ever been said upon this subject except the advocation of nitrate of silver. This seemed to be objectionable, because it discolored the teeth. Dr. Howland stated that he had used with great success tannic acid, which was the active principle of all vegetable astringents. His method was to place a drop or two of glycerin on a slab and mix it with tannic acid. He then sharpened a stick to a flat blunt point, and after drying the sensitive point to be treated he applied the mixture and with the stick rubbed very gently at first, gradually increasing the pressure until finally he was able to rub the tooth as hard as he wished without any pain whatever. One application was usually sufficient. Although a remedy was seldom found that would never fail, Dr. Howland could not remember an instancee where this method had failed to give relief. In reply to a question of Dr. F. Milton Smith, as to how long this relief was expected to last, Dr. Howland had never noted a recurrence of the sensitiveness. He not only used it to reduce this sensitiveness where there was no real decay of the tooth-structure, but he also used it as an obtundent in the same way.

In reply to a question from Dr. S. E. Davenport as to whether this pressure with the stick was necessary, Dr. Howland had never tried it any other way, as it had seemed very effective when rubbed with the stick.

Under the same head, Dr. T. W. Onderdonk stated that he had several things which he wished to present. First, a set of scalers (Fig. 1), which were no more nor less than heavy enamel chisels, one right, one left, and one direct, all curved, with concave cutting edges which act very much as half-round scrapers do in scraping the mast of a boat.

Second, a compromise dental engine arm, which consists of a duplex spring (Fig. 1), with which most cable engines are equipped, a hand-piece attached to one end, and a small pulley wheel to the other (*c*). This is attached to the forearm of any all-cord engine at *cd*, giving all the freedom of the cable engine, together with the direct revolution of the all-cord engine, without the disagreeable features of either.

Third, an electric crown heater (Fig. 2, *a*), an instrument, as its name implies, for softening the gutta-percha cement of crowns and bridges so attached, when their removal is desired. It is a curved strip of metal with some high-heat gutta-percha on the inner surface, attached to one end of the electric tool holder. To use it, bend the metal so that it will pass over the crown to be removed, turn on the current, and in a few seconds the crown can be taken off.

Fourth, Dr. Onderdonk stated that he considered the following general features essential for an ideal matrix. The matrix should grasp the tooth tightly at the cervical margin and at the same time restoring the natural contour. It should be easily adjusted and not interfere with the introduction of the filling, not easily displaced but removable at will, permitting the examination of the filling as it progresses, and be returned to its original position.

He then presented a band dentometer, contour matrix and holder. The apparatus consisted of two parts, band and holder.

Fig. 1.

The bands (Fig. 2, *b*, *c*) are cut from strips of very thin metal, either brass or German silver, two inches long and the width the case requires. For a dentometer the bands are cut straight (Fig. 2, *b*). For a matrix the bands are cut curved, the greater the curve the larger the contour (Fig. 2, *c*). The holder consists of three parts. The movable jaw, or vice, the fixed jaw, or holder, and the rotary handle. The movable jaw (Fig. 2, *d*) is of brass about one and a half inches long and three-eighths of an inch in diameter, with a hole through the centre for the introduction of the fixed jaw and a slot for the bands to enter.

The fixed jaw, or holder (Fig. 2, *e*), is about three inches long and one-eighth of an inch in diameter, with a screw thread cut

Fig. 2.

almost the entire length; on one end is the grip, which is very much like a small pair of tape pliers, containing two pins which grasp the metal bands. The rotary handle (Fig. 2, *f*) is about the same diameter as the movable holder, and is from one-half to three-quarters of an inch in length; this turns on the screw thread of the fixed jaw.

Dr. S. E. Davenport thought that Dr. Onderdonk was getting himself into trouble, as those who were not fortunate enough to sit near to him during these demonstrations would be besieging him at his office, where, as Dr. Davenport had found recently, Dr. Onderdonk was able to explain the many advantages of the appliances in even a better manner than he had done to-night.

Dr. Davenport would like to ask, in the case of a combination gold and amalgam stopping such as Dr. Onderdonk mentioned, where the gold was applied before the amalgam had crystallized, whether Dr. Onderdonk depended upon separate anchorage for the gold, or did he expect a union between the gold and amalgam sufficient to hold the gold? Dr. Davenport said he would also like to know what gold and what amalgam Dr. Onderdonk used.

Dr. Onderdonk stated that it was not his intention to enter into the discussion of the technique of filling teeth, and he merely had mentioned this method to bring out the value of his appliance. His preference was to fill the amalgam portion at one sitting and the gold afterwards, when the amalgam was hard. But if for any reason it was impracticable, the gold could be introduced at once. In either case he treated the gold filling exactly as if it, the amalgam, were tooth-structure. He used Fellowship alloy and Moss fibre gold usually, annealing it in the electric furnace. If the amalgam had not set, he used the first pieces of gold small, forcing them into the amalgam. In certain cavities in bicuspids it was his custom always to use amalgam with the gold. In these cases he preferred the amalgam soft, forcing the gold into it.

Regarding the central that he mentioned, which he filled with amalgam posteriorly, Dr. Onderdonk stated that in giving the operation in detail of course he would use a lining of cement next the anterior enamel, to prevent discoloration. He considered this method good practice in certain cases, rather than to resort to a crown. He never crowned a central tooth if he could avoid it.

Dr. Chas. O. Kimball, regarding various devices, wished to speak first of Dr. Onderdonk's appliance. He had had a couple made some time ago in accordance with Dr. Onderdonk's plans, but he found upon examination that they were not like these. These were much better. He found them exceedingly useful instruments. They were of great value in inserting a composition filling where considerable pressure was desired. Such a matrix could be removed without placing any strain on the filling. He had used them frequently in the amalgam and cement stoppings.

Dr. Kimball also spoke of a trifling little device which he had found almost invaluable. Many of those present used for polishing teeth the little soft rubber cups. If their experience had been like his, they had been found very satisfactory for that purpose, as by this appliance it was possible to polish under the free margin of the gum. This was important in order to polish the teeth thoroughly. These little rubber disks will curve and work up under the margin of the gum on the rounded surfaces of the teeth that are accessible with this instrument. Dr. Kimball had found this difficulty: in a wet mouth the saliva would work back and get into the hand-piece, so that it required to be frequently taken apart and cleaned. It had occurred to him some time ago that this could be obviated by reversing one of the cup-shaped disks and placing it on the shank of the instrument. This worked very well, but with the curious result that when it was revolving the slightest touch would cause it to work right away from the hand-piece and up to the point of the instrument. At first he had cut a groove on the shank and slipped the disk into that, but that had not proved quite strong enough, although it was an improvement. Recently he had soldered, with soft solder, a ring of wire on the shank. This worked perfectly.

Dr. Kimball called attention to the hickory sticks for holding the gum back in cervical operations. He had mentioned this method two years previously. Since that time he had been using another device of the same nature. Instead of the hickory he used a piece of thin steel cut and shaped to fit the individual case and then soldered with soft solder to a broken instrument. This could be cut carefully to fit the curve of the tooth and the gum, holding back the gum with the least possible pain.

Dr. Kimball also presented a very useful instrument for polishing fillings, particularly in concave surfaces that could not be reached with the strips or disks. It consisted of a long instrument, thin and flat at each end, one end charged with corundum, held to the instrument with shellac, and the other end carrying a small piece of Lake Superior stone held in the same manner.

Dr. Gillett, referring to the instrument mentioned by Dr. Kimball for holding the gum back, thought that in many instances an instrument not so long, and that could be held by placing the finger on the end of it, was of great advantage. Also, instead of having the end broad and flat, it was sometimes desirable to have it terminate in points, even a single point being oftentimes desirable.

Dr. F. Milton Smith wished to disclaim originality regarding the gold inlays credited to him by Dr. Onderdonk. As he had previously stated, the method was brought to his attention first by Dr. Rheinhold, of this city. Dr. Dwight Smith, of this city, had also given him some valuable ideas. His own work in this direction was the result of the combined ideas of these two gentlemen and others, with perhaps a trifle of ingenuity on his own part. His friend Dr. Rheinhold stated to him that Dr. Andrews, of New York City, first called his attention to these tips. As Dr. Smith remembered it, the specimen in the cast passed around, *combining* the abraded surface repair and the two approximal surface restorations, was first suggested by Dr. Dwight Smith.

Dr. Kimball stated, relative to the union between gold and amalgam that he had occasion, many years ago, to repair an old amalgam filling with gold. He had done this with no thought of a union between the two, but had prepared a natural retention for the gold. Some time after, the tooth had broken and the whole filling came out. He was surprised to find that although the amalgam filling had been inserted many years before the gold, the two had become firmly united, so that they could be separated with great difficulty.

Dr. Gillett had great faith in these combinations, and used the gold and amalgam fillings to a large extent in favorable cavities. It was his practice always to pack the gold directly on the fresh amalgam, believing that he got a better filling thereby than by doing the operation in two sittings.

Dr. S. E. Davenport thought Dr. Gillett's remarks proved that Dr. Dwight Clapp's influence in New York City was very strong still, and he was very glad of it.

Dr. J. Morgan Howe called attention to a paper by Professor R. H. Thurston, of Cornell University, on "Scientific Research," published in *Science,* September 12, 1902, in which he stated that the late Roberto Austin, an English metallurgist, had placed lead and gold in contact, "and later found that the molecules of gold had started off on a journey independently, into the lead, some of them reaching a distance from their original positions of two inches in as many years." Dr. Howe thought this item worth recording in this connection as throwing some light on the peculiar results observed in combining gold with tin, and gold with amalgam, in fillings.

Dr. H. L. Wheeler read a short paper entitled "Home Manufacture of Formaldehyde for Sterilizing Purposes."

(For Dr. Wheeler's paper, see page 364.)

Dr. Henry W. Gillett read a short paper on "Perplexities in Connection with Extraction of Teeth."

(For Dr. Gillett's paper, see page 368.)

Dr. Swift stated that he had been using this method of sterilization for some time in the form of the Low sterilizer. It had given great satisfaction. The apparatus was manufactured by the Buffalo Dental Manufacturing Company. The sterilizing agent was produced by a cone of sponge platinum fitted over the alcohol lamp. The shape of the instrument was very satisfactory.

Dr. Gillett agreed with Dr. Swift regarding the Low sterilizer. He thought it a very satisfactory apparatus, although he could see no reason why the one devised by Dr. Wheeler would not be of value. Regarding the other part of the paper, Dr. Wheeler argues that the dental profession has suffered greatly from dental patents; *ergo,* there should be no dental patents. Great injury had resulted from the misuse of drugs, but for that reason it would hardly be wise to give up drugs entirely. It would seem that Dr. Wheeler's statement, that the result of patent litigation was always in favor of the organized corporation, was one of the best possible arguments in favor of Dr. Crouse's organization, which Dr. Wheeler is refusing to support. By doing this he was refusing to take the one essential step to make effective the opposition to *unjust* dental patents. It was the unfair dental patents that were objectionable, and it was time now for the profession to rally and make effective the means of opposing these unjust patents. The opportunity should be taken advantage of promptly, lest it be lost.

Dr. Wheeler stated that he was acquainted with the Low sterilizer, but that the cone, instead of being sponge platinum as stated, was really made of platinized asbestos, and required frequent renewal.

Dr. H. W. Gillett read a paper entitled "Spalding's Porcelain Jacket Crown."

(For Dr. Gillett's paper, see page 362.)

Dr. McNaughton presented to the museum of the Institute some curios in the way of teeth carved from ivory, and the superior maxilla of a "sheep's-head" fish. These were donated by Dr. Nash.

A vote of thanks was extended to Dr. Nash for his kindness in presenting these interesting specimens to the Institute.

Dr. Fossume presented an engine attachment for carrying wooden points, used in cleansing and polishing teeth.

Adjourned.

Fred. L. Bogue, M.D., D.D.S.
*Editor The New York Institute of Stomatology.*

## Figures and Tables

**Figure f1:**
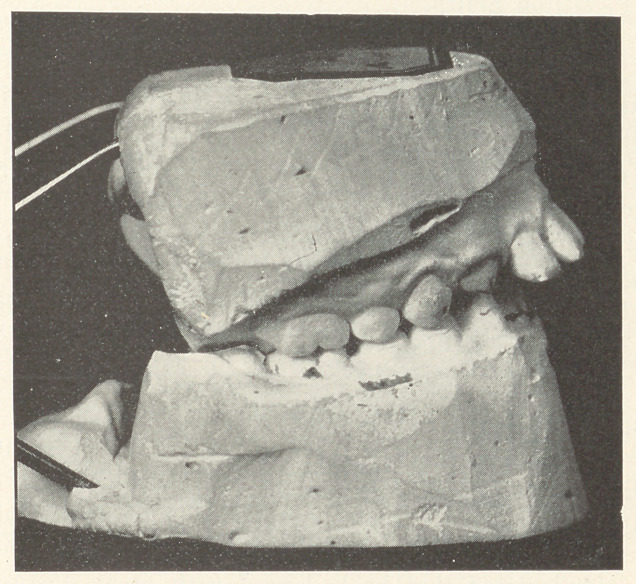


**Fig. 1. f2:**
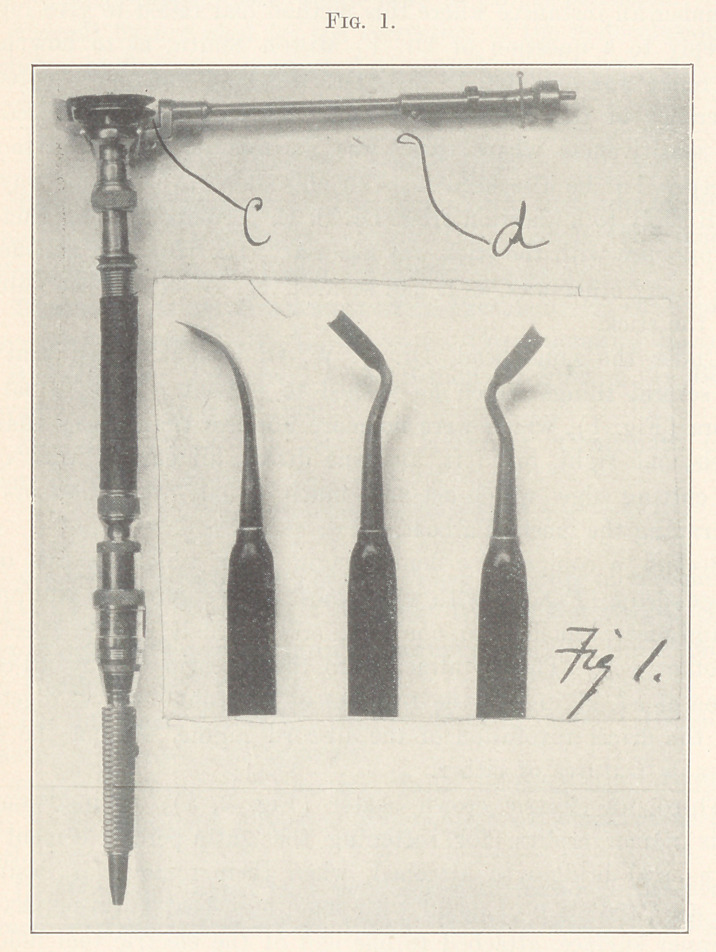


**Fig. 2. f3:**